# Laparoscopic surgery-associated massive subcutaneous emphysema requiring mechanical ventilation in a patient with endometriosis: a case report

**DOI:** 10.1093/jscr/rjac110

**Published:** 2022-03-26

**Authors:** Hideaki Tsuyoshi, Daisuke Inoue, Yumiko Miyazaki, Hiroshi Kawamura, Toshimichi Onuma, Tetsuji Kurokawa, Yoshio Yoshida

**Affiliations:** Department of Obstetrics and Gynecology, University of Fukui, Fukui, Japan; Department of Obstetrics and Gynecology, University of Fukui, Fukui, Japan; Department of Obstetrics and Gynecology, University of Fukui, Fukui, Japan; Department of Obstetrics and Gynecology, University of Fukui, Fukui, Japan; Department of Obstetrics and Gynecology, University of Fukui, Fukui, Japan; Department of Obstetrics and Gynecology, University of Fukui, Fukui, Japan; Department of Obstetrics and Gynecology, University of Fukui, Fukui, Japan

## Abstract

Although subcutaneous emphysema is a common benign complication of laparoscopic surgery, airway obstruction can occur due to pharyngeal emphysema when it extends to the neck. Here, we report a case of subcutaneous emphysema extending to the neck that required mechanical ventilation in a 51-year-old patient with endometriosis and severe adhesions during total laparoscopic hysterectomy and bilateral salpingo-oophorectomy. Although surgical or disease-specific risk stratification has not yet been established, the severe adhesions due to endometriosis and massive peritoneal defect due to the procedure might lead to the fragility of the subcutaneous tissue, resulting in a massive subcutaneous emphysema. This study highlights the importance of preoperative risk assessment in addition to intraoperative and postoperative monitoring for ventilation disorders and subcutaneous emphysema.

## INTRODUCTION

Subcutaneous emphysema is a common complication of laparoscopic surgery with an incidence rate of 0.43–2.34%. Although ~77% of patients who undergo laparoscopic surgery have grossly undetectable subcutaneous emphysema, airway obstruction can occur due to pharyngeal emphysema when it extends to the neck [1]. Although surgical or disease-specific risk stratification has not yet been established, prolonged operative time and increased number of cannulas have been associated with subcutaneous emphysema. Here, we report a case of subcutaneous emphysema extending to the neck that required mechanical ventilation in a patient with endometriosis and severe adhesions during total laparoscopic hysterectomy and bilateral salpingo-oophorectomy.

## CASE REPORT

An otherwise healthy 51-year-old woman (Gravida 2 and Para 2) visited our hospital for chronic severe dysmenorrhea. Anthropometric examination revealed a body mass index of 21.4 (weight = 47.8 kg; height = 149.4 cm). Pelvic magnetic resonance imaging demonstrated multiple uterine myomas with a 9- and 3-cm endometrioma in the right and left ovaries, respectively, prompting a total laparoscopic hysterectomy and bilateral salpingo-oophorectomy.

**Figure 1:**
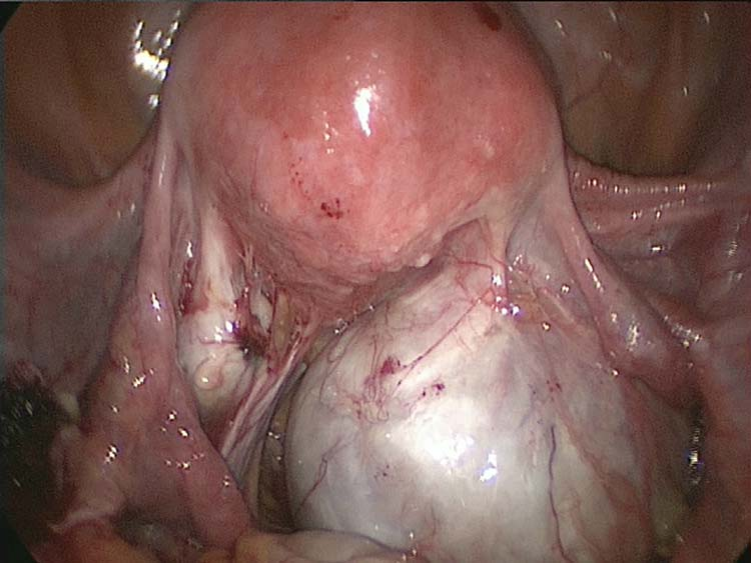
A large right ovarian cyst adherent to the uterus and retroperitoneum was identified within the Douglas cavity.

**Figure 2:**
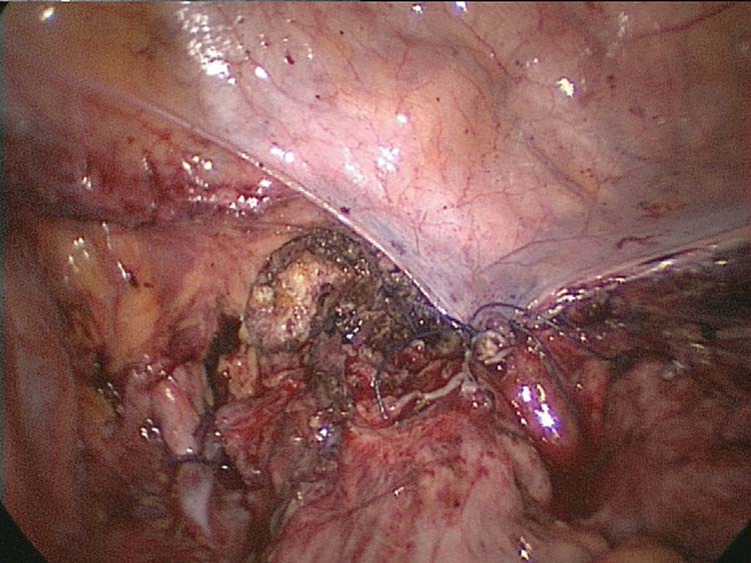
The peritoneum was massively disrupted such that the upper and lower peritoneum could not be sutured.

**Figure 3:**
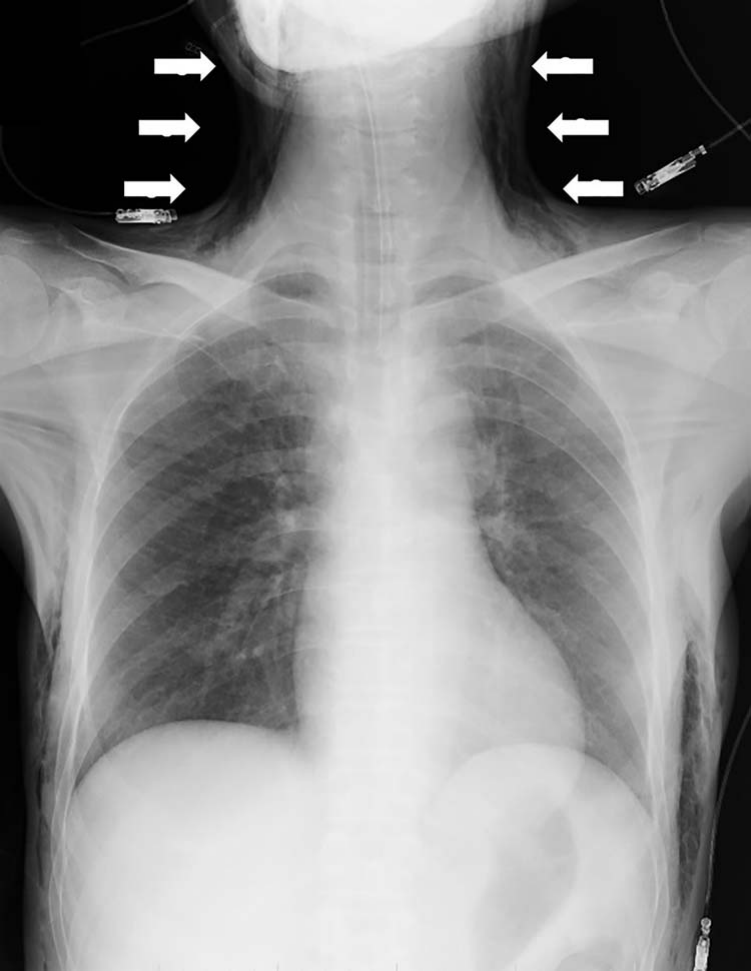
Chest radiography demonstrating the progression of the subcutaneous emphysema to the mandible (arrows).

Although in Trendelenburg position, the patient was intubated and placed under general anesthesia. A uterine manipulator was fixed on the cervix to enable uterine mobilization. A 5-mm trocar was inserted through the umbilicus. The abdomen was insufflated with CO_2_ at a maintenance pressure of 12 mmHg. Two 5-mm trocars were placed on each side of the lower abdomen. A 12-mm trocar was then inserted in the middle of the lower abdomen. Intraoperatively, a large right ovarian cyst adherent to the uterus and retroperitoneum was identified in the pouch of Douglas (Fig. 1). Several endometriotic spots were observed in the pelvic cavity. Moreover, the pouch of Douglas was partly closed with the adhesions. These findings were consistent with the diagnosis of endometriosis Stage IV based on the revised American Society for Reproductive Medicine (r-ASRM) classification [2]. However, after 1 h, the patient presented with subcutaneous emphysema extending the anterior chest wall with an associated increase in end-tidal CO_2_ from 40 to 50 mmHg. Reducing the insufflation pressure from 12 to 8 mmHg and increasing the frequency of ventilation improved the patient’s condition. Peeling off those adhesions, the total laparoscopic hysterectomy and bilateral salpingo-oophorectomy were performed without complications. However, the peritoneum was massively disrupted such that the upper and lower peritoneum could not be sutured (Fig. 2). The total operation time was 3 h and 49 min. Pre-extubation chest radiography showed progression of the subcutaneous emphysema to the mandible in the absence of pneumothorax or pneumomediastinum (Fig. 3). This prompted close observation and continuation of mechanical ventilation. After 24 h, the subcutaneous emphysema of the neck was reduced and the patient was extubated. On the sixth postoperative day, the subcutaneous emphysema was completely resolved and she was discharged without any complications.

## DISCUSSION

Subcutaneous emphysema is a common complication of laparoscopic surgery caused by the extravasation of CO_2_ into the subcutaneous tissues. Although most cases are mild, airway obstruction can occur due to pharyngeal emphysema when the neck is involved. Moreover, in cases of massive subcutaneous emphysema, CO_2_ in the subcutaneous tissue can diffuse into the blood, resulting in severe hypercarbia [1, 3].

Risk factors for subcutaneous emphysema (e.g. improper cannula placement, use of more than five cannulas, increased intra-abdominal pressure, operative time more than 3.5 h, age > 65 years and low body mass index) have been reported [1]. However, surgical and disease-specific risk stratification models have not yet been established.

Murdock CM *et al*. reviewed the predictors for subcutaneous emphysema in various types of surgery (i.e. gynecologic procedures, Nissen fundoplication, hernia repair, cholecystectomy and all other general surgeries). However, the type of surgery was not significantly associated with subcutaneous emphysema [3]. Lee *et al*. conducted a prospective randomized study to estimate the risk factors related to the occurrence of subcutaneous emphysema during laparoscopic gynecologic surgery. Surgeries that required intra-abdominal suturing (e.g. total hysterectomy) had a higher incidence of subcutaneous emphysema than in procedures that do not require intra-abdominal suturing (e.g. ovarian cystectomy). The indications for surgery were myoma, adenomyosis, benign adnexal disease, vault prolapse, cervical intraepithelial neoplasia and endometrial hyperplasia. However, disease-specific risk stratification has not been discussed [4].

To the best of our knowledge, only a few reports of massive subcutaneous emphysema with hypercarbia that required mechanical ventilation have been reported in patients who underwent laparoscopic gynecologic surgeries [5–8]. The types of surgery were supra-cervical hysterectomy, total laparoscopic hysterectomy and salpingo-oophorectomy. Moreover, the surgical indications were myoma, cervical carcinoma *in situ* and ovarian tumor. Among these patients, two cases experienced pneumomediastinum or pneumopericardium. In both cases, the operative time was >200 min. Although the prolonged operative time may have precipitated the massive subcutaneous emphysema, the impact of patient factors, type of surgery and indication for surgery is still unclear.

Endometriosis refers to the presence of endometrial glands or stroma in sites other than the uterine cavity. The pathogenesis of endometriosis involves the interaction of endocrinologic, immunologic, pro-inflammatory and pro-angiogenic processes, leading to severe adhesions, dysmenorrhea and infertility [9]. Although strong evidence supports the utility of laparoscopic surgery in improving pain relief and fertility, its association with subcutaneous emphysema remains unclear [10].

In the present case, she had r-ASRM Stage IV endometriosis, which required peeling off of the adhesion and extensive resection of the peritoneal endometriosis. Therefore, the massive subcutaneous emphysema is caused not only by the prolonged operative time but also by the fragility of the peritoneal tissue due to the peritoneal endometriosis and extensive resection.

In conclusion, this study highlights the importance of considering the risk of massive subcutaneous emphysema during laparoscopic surgery in patients with chronic peritoneal inflammatory diseases (e.g. endometriosis).
